# Evaluation of a Russian version of the oral health literacy instrument (OHLI)

**DOI:** 10.1186/1472-6831-14-141

**Published:** 2014-11-27

**Authors:** Anastasiya Blizniuk, Masayuki Ueno, Sayaka Furukawa, Yoko Kawaguchi

**Affiliations:** Department of Oral Health Promotion, Graduate School of Medical and Dental Sciences, Tokyo Medical and Dental University, Tokyo, Japan

**Keywords:** Oral health literacy, Oral health literacy instrument (OHLI), Russian version, Validation studies

## Abstract

**Background:**

Oral health literacy has become a popular research area in the last decade; however, to date no health literacy instruments in the Russian language exist. The objectives of this study were to develop a Russian version of the Oral Health Literacy Instrument (OHLI) and to examine its reliability and validity.

**Methods:**

A convenience sample of patients who visited the dental division of the district hospital in Belarus was used in the study. The OHLI, created originally in English, was modified to adapt it to characteristics of routine dental services in Belarus and then translated into Russian, followed by back-translation. Participants completed a self-administered socio-demographic questionnaire, an oral health knowledge test and the Russian version of the OHLI (R-OHLI). Bivariate and multivariate statistical analyses, including multiple regression modeling, were performed to examine reliability and validity of the R-OHLI.

**Results:**

Participants were 281 adult patients aged from 18 to 60 years, with a mean age of 33.1 ± 12.2; 64.1% of them were women. Cronbach’s alpha values for the two sections (reading comprehension and numeracy) and the total R-OHLI were 0.853, 0.815 and 0.895, respectively. The mean total R-OHLI score was 77.2 ± 14.5; the mean reading comprehension and numeracy scores were 39.5 ± 7.5 and 37.8 ± 8.8, respectively. The R-OHLI was significantly correlated to the oral health knowledge test. Pearson’s correlation coefficients between the oral health knowledge test and the reading comprehension, numeracy and total R-OHLI were 0.401, 0.258, and 0.363, respectively (*p* < 0.001). Women, participants with a university degree, and those who visited a dentist at least once a year had significantly (p < 0.05) higher mean scores for each section (reading comprehension, numeracy) and for total R-OHLI compared to their counterparts.

**Conclusions:**

The R-OHLI showed good internal consistency and test-retest reliability. It was significantly associated with the oral health knowledge test, socio-demographic and behavioral factors. Therefore, the R-OHLI was proved to be a reliable and valid oral health literacy instrument for Russian-speaking people.

## Background

The concept of health literacy has received significant research attention in the last decade. Health literacy is of particular importance when dealing with chronic conditions and life-style diseases [1, 2]. Oral diseases are highly preventable, but they remain common in many countries throughout the world [3]. For oral health literacy, the definition of health literacy developed by Ratzan and Parker [4] was later adopted for use in the context of oral health. Oral health literacy is defined as the “degree in which individuals have the capacity to obtain, process and understand basic oral health information and services needed to make appropriate health decisions”[5]. Oral health literacy is a complex concept, and its functional aspect can be described as a set of personal skills and abilities that enable oral health related knowledge acquisition and decision-making. For that reason, high oral health literacy is essential to improve people’s awareness of oral disease, knowledge about methods of disease prevention and health maintenance, and to eventually lead people to desirable attitudes and behaviors.

Previous findings demonstrated that low oral health literacy was widespread and could explain certain inequalities in oral health [6–14]. A number of studies showed that limited oral health literacy contributes to poor oral health status [10, 14]. People with lower oral health literacy are more likely to neglect prevention measures and seek emergency treatment [12]. Parents’ or caregivers’ oral health literacy affects children’s oral health outcomes [15, 16]. The majority of studies on oral health literacy have been conducted in North America [8–10, 12, 13, 15–17] and some Asian countries [11, 14, 18, 19]. The European office of the WHO reported in 2013 that nearly half of Europeans had “inadequate or problematic health literacy skills”, but with great variance among countries [20]. These data suggest that oral health literacy levels in Europe may not be high; however, no systematic research data on oral health literacy are available [21]. The scarcity of oral health literacy information requires more research to be done in these countries.

Oral health related jargon is specific and differs from the terms used in general medicine, leading to the development of specific tests measuring an ability to understand dental terms and oral health related information. Further, because the majority of studies on oral health literacy were produced in the USA, existing oral health literacy assessment instruments are predominantly in English. There are two main strategies to evaluate oral health literacy: word recognition and reading comprehension. Word recognition instruments (REALD-30, REALD-99, REALM-D, TS-REALD, HKREALD-30) [7, 18, 22–24] were created first and concentrated on a respondent’s ability to correctly pronounce oral health related vocabularies. Reading comprehension tests, such as the TOFHLiD, OHLI, OHL-AQ, HKOHLAT-P [17, 18, 25, 26] were constructed to evaluate functional literacy, and, therefore, measured a person’s ability to understand and apply written information, including numerical data. Macek’s comprehensive oral health knowledge test (CMOHK) [13] aimed to assess oral health literacy levels through measurement of conceptual oral health knowledge. Quite a large number of instruments exist, but none of them is adequate to measure all dimensions of oral health literacy. Although most were developed in English [13, 17, 22–25], several oral health literacy instruments are available in Spanish, Persian and Chinese [18, 19, 26, 27]. However, to date there is no health literacy instrument in Russian, which is the native language for 162 million people living in 16 countries [28].

It is difficult to apply English word-recognition instruments to other languages due to certain linguistic differences [27]. For that reason we chose reading comprehension as a method for oral health literacy assessment. At the time of developing the study design, only two reading comprehension instruments existed: the OHLI [17] and the TOFHLiD [25]. The OHLI was chosen to develop an instrument for Russian speakers because of its better reliability and validity than the TOFHLiD [17, 25]. Moreover, the cloze procedure, on which the OHLI is based, has been widely used and proved to be effective for reading comprehension assessment in Russian [29].

The OHLI is a validated functional literacy test, measuring not only reading comprehension but also numeracy ability [17]. The objectives of this study were to develop a Russian version of the OHLI and to examine its reliability and validity.

## Methods

### Subjects

The study was conducted using a convenience sample of adult patients who visited the dental division of the district hospital in Belarus during July and August of 2013. Adult patients aged 18-60 years, and without physical or mental disabilities, were asked to participate in this study. A written informed consent was obtained from each participant. Patients older than 60 years were excluded to create a homogenous sample. We were granted permission to conduct the survey by the hospital’s administration. The research protocol was also approved by the Tokyo Medical and Dental University Ethical Committee prior to data collection (Approval No 901).

### Questionnaire

A self-administered questionnaire was used to collect information about socio-demographics (gender, age, education – junior high school, high school or university level) and oral health behavior (regularity of dental visits – less than once a year or once a year and more).

### Oral health knowledge test

The oral health knowledge test applied by Sabbahi, et al. to validate the OHLI [17] was created in accordance with the information used in education materials in Canada. There were certain difficulties in its application to the Belarusian population, because the prevalence of oral disease and oral health conditions, as well as routine dental treatment procedures, in Belarus differed significantly. For that reason, we developed a new oral health knowledge test that followed the actual oral health situation and referred to oral health related materials in Belarus. The oral health knowledge test used in the study included 10 statements regarding dental caries, periodontal disease, oral cancer and oral hygiene (Table 1). Subjects were asked to score each statement as true or false; correct answers were scored with 1, incorrect or don’t know answers were scored with 0. Total oral health knowledge scores ranged from 0 to 10 (the sum of scores for each item). Oral health knowledge test’s scores were multiplied by 10 to create a weighted scale from 0 to 100 (100/10).

**Table 1:** **Oral health knowledge test**

1	Dental decay is caused by the bacteria of the oral cavity	true	false	don’t know
2	Sweet food and drinks have positive effect on the teeth	true	false	don’t know
3	Use of fluoride makes the teeth stronger	true	false	don’t know
4	Sealants are dark spots on the teeth	true	false	don’t know
5	Dental plaque causes periodontal diseases	true	false	don’t know
6	There is no relationship between periodontal diseases and diabetes	true	false	don’t know
7	It is necessary to use a dental floss every day to clean between the teeth	true	false	don’t know
8	The teeth should be brushed at least twice a day	true	false	don’t know
9	Cancer cannot appear in the oral cavity	true	false	don’t know
10	Visiting a dentist once a year helps to preserve oral health	true	false	don’t know

### R-OHLI

The R-OHLI is a Russian-language version of the OHLI, created originally in English by Sabbahi, et al. (2009). The OHLI is a functional oral health literacy test based on the TOFHLA [30], and consists of cloze-procedure based reading comprehension and numeracy sections. The reading comprehension section has 38 items and assesses the individual’s ability to read and understand written information about dental disease. Words were omitted from 2 passages, one on dental caries and the other on periodontal disease. The numeracy section has 19 items and evaluates the individual’s ability to comprehend common dental medication prescriptions, dental appointments and instructions that require performing some basic mathematical operations [17].

First, the English version of OHLI was modified to suit routine dental services in Belarus. Only minor modifications of content were made in order to preserve the instrument’s original structure of the passages, including the number of sentences. We changed a few sentences of the reading comprehension section because amalgam fillings are not used in Belarus. In the numeracy section a medication prescription outline was changed to meet the requirements of Belarusian practice. The number of items in each R-OHLI section is the same as that of the OHLI. The Russian version also met the criteria of the cloze procedure [31]. Similar to the OHLI, four possible answers were given for each omitted word. One of these was correct while the others either sounded similar or were grammatically incorrect [17].

The modified English OHLI version was then translated into Russian by one of the authors (a native Russian speaker), following by back-translation (made by an independent translator with high proficiency in both Russian and English languages). Two translators evaluated the equivalence between the original and back-translated versions, and they concluded that the quality of translation was good.

No time limitation was set for completing the R-OHLI. The R-OHLI items were scored in the same way as the original OHLI [17]; each item was scored with 1 if correct or 0 if not correct or unanswered. The final scores for each section were a sum of each item. As with the original OHLI [17], the scores of the reading comprehension and numeracy sections were multiplied by 1.316 (50/38) and 2.632 (50/19), respectively. Hence, possible weighted scores of each section ranged from 0 to 50, and the possible total R-OHLI score ranged from 0 to 100 (the sum of the reading comprehension and numeracy sections).

We obtained permission from D. Sabbahi to translate the OHLI into Russian language. The R-OHLI is available upon request from the corresponding author.

### Statistical analysis

Statistical analyses included bivariate and multivariate methods. The readability level of the R-OHLI reading comprehension section was evaluated with Flesch Reading Easy (FRE) scores [32], modified for the Russian language [33].

Internal consistency was assessed with Chronbach’s alpha. Ten participants, who agreed to attend the hospital one more time for retesting, completed the R-OHLI once again two weeks after their first visit. The intra-class correlation (ICC) was used to evaluate test-retest reliability.

Concurrent validity (how well a test has an ability to distinguish between characteristically different groups [34]) was verified by comparing the R-OHLI scores between groups with different socio-demographic and oral health behavioral characteristics using independent *t*-tests. To verify construct validity (the degree to which a test measures what it is supposed to measure [34]) a Pearson’s correlation coefficient between the R-OHLI and the oral health knowledge scores was calculated. Further, an association of the R-OHLI with other variables was analyzed using multiple linear regression. Two multiple linear regression models were built with the R-OHLI scores as an outcome variable. Selection of variables was based on a study by Sabbahi, et al. [17], and we included the same variables in the model to compare between the English and Russian versions’ validation results. At the first step, education level and regularity of dental visits were included in the model, besides controlling for age and gender (Model 1). Next, the oral health knowledge test score was added to the model (Model 2).

SPSS 17.0 (IBM Inc.) statistical package was used for all statistical analyses.

## Results

### Socio-demographics

All data from subjects who agreed to participate in the study were used for the analysis. In total, 281 participants with a mean age of 33.1 ± 12.2 years were included (Table 2). Among participants, 64.1% were women; mean ages of females and males were 35.9 ± 12.3 and 28.0 ± 10.3 years, respectively.

**Table 2:** **Sample characteristics of the study subjects**

	***n*** = 281
Age, years	
Mean age (SD)	33.1 (12.2)
Gender, % (*n*)	
Male	35.4% (101)
Female	64.1% (180)
Education level, % (*n*)	
High school or less	75.4% (212)
University or higher	24.6% (69)
Regularity of dental visits, % (*n*)	
Non-regular (less than once a year)	48.4% (136)
Regular (once a year or more)	51.6% (145)

As for education, 69 (24.6%) respondents had a university or postgraduate degree, 204 (72.6%) graduated from high school and 8 (2.8%) participants had only a compulsory, junior high school education. The respondents were divided into two education level groups: with and without a university degree. There was no significant distributional difference in the education level by gender; 26.1% of female and 21.8% of male participants had a university diploma. The mean ages of participants with and without a university education were 35.3 ± 11.3 and 32.3 ± 12.4 years, respectively, and were not significantly different.

### Oral health behavior

People who visited a dentist once a year or more were considered to be regular dental visitors. The proportions of participants who visited a dentist regularly and those who did not were almost equal; 51.6% of subjects visited a dentist on a regular basis. The mean ages of regular and non-regular dental visitors were 33.4 ± 12.7 and 32.8 ± 11.7 years, respectively, and they were not significantly different. However, female participants visited a dentist significantly more regularly than males; 58.9% of females and 38.6% of males visited a dentist at least once a year (*p* < 0.01).

### Oral health knowledge test

The mean oral health knowledge score was 63.8 ± 16.9. Oral health knowledge was significantly associated with socio-demographic characteristics (gender, education) and regularity of dental visits (Table 3). Female participants, individuals with a university degree or regular dental visitors had significantly (*p* < 0.01) higher oral health knowledge test scores compared to their counterparts.

**Table 3:** **Mean** (±**SD**) **oral health knowledge and R**-**OHLI scores by socio**-**demographic and health behavioral characteristics**

		***n***	Oral health knowledge test (10 items)	R-OHLI reading comprehension section (38 items)	R-OHLI numeracy section (19 items)	R-OHLI total (57 items)
Gender	Male	101	59.8 ± 17.2	37.3 ± 8.3	36.3 ± 9.2	73.6 ± 15.4
	Female	180	66.1 ± 16.3	40.6 ± 6.7	38.7 ± 8.4	79.3 ± 13.6
	*p*-value		<0.01	<0.01	<0.05	<0.01
Education	High school	212	61.1 ± 16.3	38.5 ± 7.8	36.7 ± 9.2	75.2 ± 15.1
	University	69	72.0 ± 15.6	42.4 ± 5.6	41.1 ± 6.3	83.5 ± 10.0
	*p*-value		<0.001	<0.001	<0.001	<0.001
Regularity of dental visits	Non-regular	136	59.8 ± 16.3	38.4 ± 7.2	36.7 ± 9.0	75.1 ± 14.8
	Regular	145	76.6 ± 16.6	40.4 ± 7.4	38.9 ± 8.5	79.3 ± 14.0
	*p*-value		<0.001	<0.05	<0.05	<0.05

### R-OHLI

**Figure 1:**
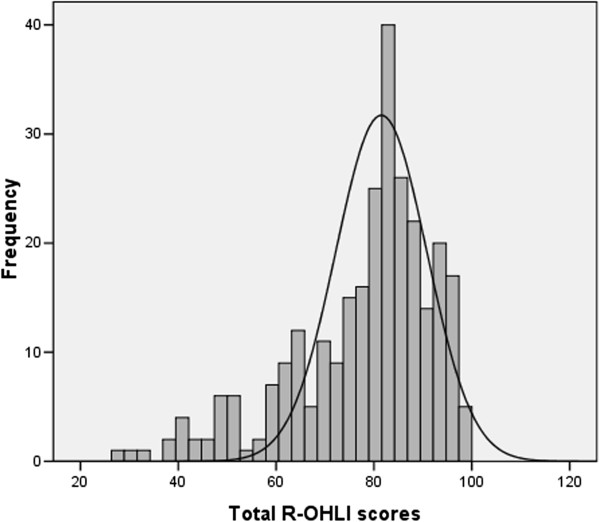
**Histogram of the distribution of the R**-**OHLI scores**.

The distribution of the R-OHLI scores was skewed a little to the left, with the majority of participants having high R-OHLI scores (Figure 1), but it did not depart far from a normal distribution (skewness -1.1, kurtosis 0.9). The mean total R-OHLI score was 77.2 ± 14.5, the mean reading comprehension and numeracy scores were 39.5 ± 7.5 and 37.8 ± 8.8, respectively.

The FRE readability scores for both the dental caries and periodontal disease passages of the reading comprehension section were higher than 30 (36.8 and 34.7, respectively).

Cronbach’s alpha scores were high: 0.853 and 0.815 for reading comprehension and numeracy sections, respectively, and 0.895 for the total R-OHLI. The intra-class correlation (ICC) values showed good agreement between test-retest results (*n* = 10). The ICC for reading comprehension was 0.899 with a 95% CI from 0.647 to 0.974. The ICC for the numeracy section was 0.637, with a 95% CI from 0.056 to 0.896, and the ICC for the total R-OHLI was 0.875 with a 95% CI from 0.577 to 0.967.

To determine concurrent validity the mean R-OHLI scores were compared by socio-demographic and health behavioral characteristics. Females, participants with a university degree or those who visited a dentist at least once a year had significantly (*p* < 0.05) higher mean scores in each section and the total R-OHLI compared to their counterparts (Table 3).

As for construct validity, the Pearson’s correlation coefficient showed that the R-OHLI was significantly (*p* < 0.001) correlated to the oral health knowledge test (Table 4). The highest correlation coefficient (r = 0.401) of the R-OHLI was with the reading comprehension section.

**Table 4:** **Pearson**’**s correlation coefficients** (**CC**) **between R**-**OHLI and oral health knowledge test**

	CC ***(n = 281)***	***p***-value
Reading comprehension section (38 items)	0.401	<0.001
Numeracy section (19 items)	0.258	<0.001
Total R-OHLI (57 items)	0.363	<0.001

The first multiple linear regression model, including socio-demographic and behavioral factors, showed the R-OHLI scores to be significantly associated with participant’s gender and education level (Table 5). Inclusion of the oral health knowledge scores in the model raised the adjusted coefficient of determination (adjusted R^2^) from 0.086 (Model 1) to 0.157 (Model 2) indicating that 16% of the variance of the R-OHLI scores was explained by Model 2. Multiple regression analysis found the R-OHLI and the oral health knowledge test to be significantly associated, even adjusting for socio-demographic and oral health behavioral factors (*p* < 0.001).

**Table 5:** **Multiple linear regression results of the R**-**OHLI scores**

	Model 1			Model 2		
	S.E.	Beta	***p***	S.E.	Beta	***p***
Age	0.1	0.0	0.980	0.1	-0.0	0.970
Gender	1.8	0.2	0.008	1.8	0.1	0.036
Education	1.9	0.2	0.000	1.9	0.2	0.008
Regularity of dental visits	1.7	0.1	0.157	1.7	0.0	0.558
Oral health knowledge	-	-	-	0.1	0.3	0.000

## Discussion

Little information is available on the oral health knowledge and literacy status of Russian-speaking populations. Currently, no health literacy instrument is available in the Russian language, which is the 5^th^ most spoken language in the world by total number of speakers and one of the six official languages of the United Nations. Thus, creation and validation of an oral health literacy instrument is the first step in any oral health literacy study among adult Russian speakers.

A considerable number of oral health literacy tools have been developed [7, 13, 17–19, 22–26], and all of them measure different aspects of oral health literacy or use diverse items. Hence, it is difficult to compare results from previously conducted studies. Furthermore, there is no consensus on what level of oral health literacy is “low” or “high”; even the REALD-30, the most widely used oral health literacy test, has no pre-established cut-off points [22].

English word recognition instruments, based on the correct pronunciation, could not be used because of the more regular phonetic structure of the Russian language. Therefore, we adapted an existing reading comprehension tool, the OHLI [17], for Russian speakers with the least possible content modification, instead of creating a new oral health literacy instument. In this paper we examined the reliability and validity of the first functional oral health literacy instrument in Russian, the R-OHLI.

The readability scores indicated that the R-OHLI reading-comprehension section’s text did not require the reader to have a university degree to understand its content, thus the readability of the R-OHLI was appropriate for an average adult. Even though scores were lower than those of the original version [13], we deemed readability of the R-OHLI satisfactory. In Belarus, the literacy rate in the general population is 99.6% [27]. Compulsory education is 9 years long, from primary to junior high school, and the average length of schooling is 16 years [27]. Therefore, reading ability was assumed to be good among Belarusians. In fact, no participants reported any problems understanding the questionnaire’s content.

In the present study, the R-OHLI showed good internal consistency by Cronbach’s alpha and test-retest reliablity by the ICC. These results were in line with reliability characterictics of the original English version of the OHLI [17]. Thus, the translation process and necessary content modifications did not significanlty alter the questionnaire’s original reliability.

Concurrent validity of the R-OHLI was proved by comparing oral health literacy scores between groups with different gender, education level and dental-visits frequency. Although previous studies did not find a significant difference in oral health literacy level between the sexes, gender was significantly associated with the R-OHLI scores in the present study, both in bivariate and multivariate analyses. The higher oral health literacy among female participants in the present study may be attributed to confounding factors that were not included in the analysis, such as health information-seeking behaviors, higher exposure to health related information during pregnancy, and frequent utilization of medical facilities with small children.

Similar to the previous studies, we found participants with a higher education to have higher oral health literacy scores [7, 9, 15–17, 22, 26, 27]. The effect of education on oral health literacy level remained significant after adjusting for age, gender, regularity of dental visits and oral health knowledge in the multivariate analysis. Regularity of dental visits was a significant factor for oral health literacy in the univariate analysis, as reported by other researches [14, 17, 22]. However, its effect disappeared after including socio-demographic factors in the multuvatiate analysis. Thus, socio-demographic factors may play a role in the relationship between oral health literacy and dental-visits pattern.

Like the original OHLI, the R-OHLI was significantly correlated with an oral health knowledge test [17]. This significant correlation can be explained by Baker’s model of health literacy [1], where conceptual health knowledge is seen as a necessary background for an individual’s health literacy.

This study proved the Russian version of the OHLI to be reliable and valid. The R-OHLI is a long questionnaire and requires significant time to be completed (about 20-30 minutes on average). For that reason its application for clinical practice may be limited. However, it was confirmed to be a useful research tool to assess the functional oral health literacy among adults.

Certain limitations should be acknowledged. To date, no health literacy instruments exists in Russian, therefore, it was not possible to analyze associations between general health and oral health literacy in this study to evaluate convergent (the degree to which two different instruments measuring theoretically related constructs are in fact related) and predictive (the extent to which an instrument predicts scores on some related criterion) validity [34]. The oral health knowledge test used to establish the construct validity was not validated, which could influence study results. Internal consistency was established with a limited number of participants, which resulted in a relatively high ICC confidence interval. In addition, the information was collected from a convenience sample of patients aged 18-60 years who visited a dental clinic. Therefore, they are likely to have higher oral health literacy than the population in general, especially with the study’s exclusion of older people. Future research using a general population sample will be necessary to examine the association of oral health literacy with oral health outcomes, to further scrutinize validity, and to determine cut-off points for the R-OHLI scale.

## Conclusions

We developed a Russian version of the OHLI and evaluated its reliability and validity on dental patients. The R-OHLI showed good internal consistency and test-retest reliability. The R-OHLI scores were significantly associated with socio-demographic and behavioral factors in bivariate analyses. Moreover, the multivariate analysis found a significant association between the R-OHLI and the oral health knowledge test results. Therefore, it was concluded that the R-OHLI is a reliable and valid oral health literacy assessment instrument for Russian-speaking people.
